# TILL-D: An *Aegilops tauschii* TILLING Resource for Wheat Improvement

**DOI:** 10.3389/fpls.2018.01665

**Published:** 2018-11-14

**Authors:** Nidhi Rawat, Adam Schoen, Lovepreet Singh, Alexander Mahlandt, Duane L. Wilson, Sanzhen Liu, Guifang Lin, Bikram S. Gill, Vijay K. Tiwari

**Affiliations:** ^1^Plant Science and Landscape Architecture Department, University of Maryland, College Park, College Park, MD, United States; ^2^Plant Pathology Department, Kansas State University, Manhattan, KS, United States

**Keywords:** *Aegilops tauschii*, D genome donor, bread wheat, TILLING, mutation frequency, genes

## Abstract

*Aegilops tauschii* (2n = 2x = 14, genome DD), also known as Tausch’s goatgrass, is the D genome donor of bread or hexaploid wheat *Triticum aestivum* (2n = 2x = 42, AABBDD genome). It is a rich reservoir of useful genes for biotic and abiotic stress tolerance for wheat improvement. We developed a TILLING (Targeting Induced Local Lesions In Genomes) resource for *Ae. tauschii* for discovery and validation of useful genes in the D genome of wheat. The population, referred to as TILL-D, was developed with ethyl methanesulfonate (EMS) mutagen. The survival rate in M_1_ generation was 73%, out of which 22% plants were sterile. In the M_2_ generation 25% of the planted seeds showed phenotypic mutations such as albinos, chlorinas, no germination, variegated, sterile and partially fertile events, and 2,656 produced fertile M_2_ plants. The *waxy* gene was used to calculate the mutation frequency (1/70 kb) of the developed population, which was found to be higher than known mutation frequencies for diploid plants (1/89–1/1000 kb), but lower than that for a polyploid species (1/24–1/51 kb). The TILL-D resource, together with the newly published *Ae. tauschii* reference genome sequence, will facilitate gene discoveries and validations of agronomically important traits and their eventual fine transfer in bread wheat.

## Introduction

Hexaploid bread wheat *Triticum aestivum* (2n = 6x = 42, AABBDD) arose by a hybridization event between cultivated emmer wheat *T. turgidum* (2n = 4x = 28, AABB) and *Aegilops tauschii* (2n = 2x = 14, DD) in the south of the Caspian Sea around 8,000 years ago (McFadden and Sears, 1946; Kihara et al., 1965; Wang et al., 2013). *T. aestivum* is a comparatively young member of the Triticeae tribe having a narrow genetic base (Dvorak et al., 1998). Enhancing the genetic diversity of wheat will be essential to cope with rapid evolution of pathogen races, changing climatic conditions, and demand for increasing crop production (Mba et al., 2012; Mondal et al., 2016). *Ae. tauschii* is an excellent source of useful genes against diseases and abiotic stresses (Gill et al., 1986b; Dhaliwal et al., 1991; Assefa and Fehrmann, 2004; Kalia et al., 2016). Genetic closeness to wheat, easy crossability and rich diversity of useful genes and alleles make it simple and convenient to use *Ae. tauschii* for wheat improvement (Gill et al., 2006). Transfer of useful genes from *Ae. tauschii* to wheat can be done either by direct hybridization or as synthetic hexaploid wheat (McFadden and Sears, 1946; Gill and Raupp, 1987). Both of these approaches have been used to transfer resistance against pathogens (Dhaliwal et al., 1991; Cox et al., 1994; Murphy et al., 1998; Singh et al., 2000), pests (Gill et al., 1986a; Xu et al., 2006), abiotic stresses (Ryan et al., 2010; Pradhan et al., 2012; Ogbonnaya et al., 2013), for quality traits (Lagudah et al., 1987; Cox et al., 1997; Brown-Guedira et al., 2005) as well as yield parameters (Mujeeb-Kazi et al., 2008; Okamoto et al., 2013).

Bread wheat is one of the most important staple food crops of the world. Due to its large genome size (∼16 × 10^9^ bp/1C, Arumuganathan and Earle, 1991; International Wheat Genome Sequencing Consortium [IWGSC], 2018), high repeat content (more than 85%, Kam-Morgan et al., 1989; International Wheat Genome Sequencing Consortium [IWGSC], 2018), and the three constituent homologous genomes, gene-discovery and map-based cloning has lagged behind other crops such as maize and rice (Rasheed et al., 2018). Diploid progenitors such as the D genome donor of wheat – *Ae. tauschii* provide a practical alternative for gene identification and cloning in wheat (Huang et al., 2003; Ling et al., 2004). The recent availability of reference-quality genome sequence of *Ae. tauschii* will speed up gene cloning in wheat, especially those coming from the D genome (Luo et al., 2017; Rasheed et al., 2018). This will open tremendous opportunities to transfer more useful genes from *Ae. tauschii* as well as their identification and molecular characterization. Genetic and reverse-genetic populations of *Ae. tauschii* will provide resources for identifying and validating useful genes with the help of its high-quality reference genome sequence. In the present work, we report the development and characterization of a TILLING (Targeting Induced Local Lesions In Genomes) resource of *Ae. tauschii*. Establishing phenotype to genotype relations and allocating function to variant alleles/ genes is comparatively easier working directly with the ‘D’ genome TILLING resource.

TILLING offers several advantages over other reverse genetics approaches because it can successfully be applied to any plant species or variety (Greene et al., 2003). Gene validation strategies such as genome-editing or RNAi-induced gene silencing are promising but limited because of transformation bottlenecks. At present, transformation efficiency of most of the wheat varieties is very low (Bhalla, 2006; Harwood, 2012). Additionally, TILLING populations provide immortal collections of variants for any gene, unlike transformation-based approaches, where every gene has to be targeted specifically (Uauy et al., 2009). TILLING has been used extensively to validate gene functions in wheat gene cloning projects (Krattinger et al., 2009; Periyannan et al., 2013; Saintenac et al., 2013; Moore et al., 2015; Rawat et al., 2016). TILLING in a number of genotypes of hexaploid and tetraploid wheat has been reported by several researchers (Slade et al., 2005; Xin et al., 2008; Dong et al., 2009; Uauy et al., 2009). Rawat et al. (2012) developed a TILLING resource in ‘A’ genome wheat *T. monococcum* as a diploid model to investigate gene functions in bread wheat. In addition to their use as reverse genetic resources for gene validation, mutagenized populations have been used for rapid cloning of disease resistance genes in plants using MutRenSeq approach (Steuernagel et al., 2016). TILLING populations also serve as resources for forward genetic screens for useful novel mutations in genes (Caldwell et al., 2004; Parry et al., 2009; Kurowska et al., 2011). In the present work, we report the development and characterization of a TILLING resource of *Ae. tauschii* as a tool for gene discovery and validation for D genome of bread wheat.

## Materials and Methods

### Plant Material and EMS Mutagenesis

**FIGURE 1:**
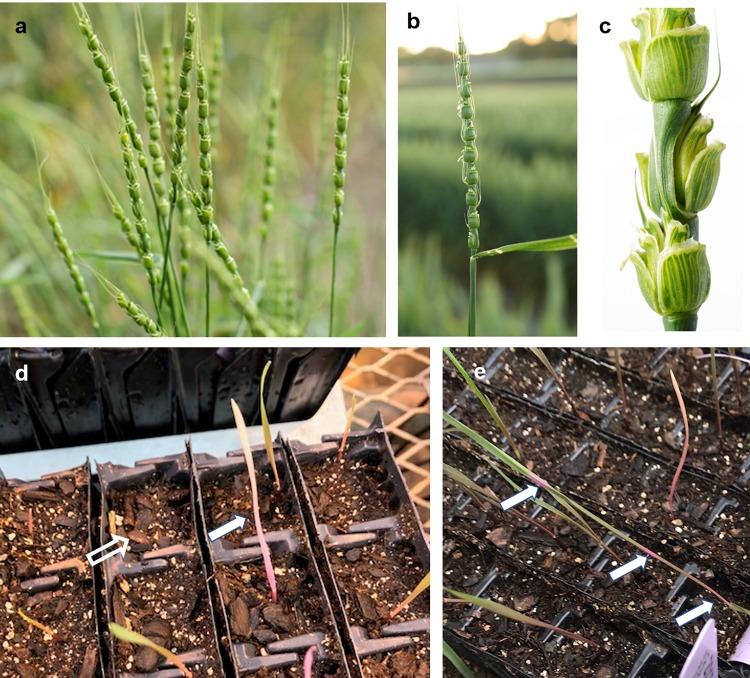
Spikes of wild type *Aegilops tauschii* subsp. *strangulata* and some phenotypic mutant seedlings. **(a,b)** Spikes of *Ae. tauschii* subsp. *strangulata* wild type plants, **(c)** close-up of a some spikelets of a wild type spike, **(d)** an albino mutant (marked with a solid arrow), and no germination (marked with a hollow arrow), **(e)** a variegated mutant seedling showing bands of pink coloration (indicated with arrows) on the leaf.

*Aegilops tauschii* subsp. *strangulata* (WGRC accession number TA 2450) was used to develop the TILL-D TILLING population. Figure 1 shows some pictures of wild type *Ae. tauschii* subsp. *strangulata* plants and spikes. Seeds were manually peeled from the tough spikes of *Ae. tauschii*. Accession TA 2450 is a winter-type genotype originally collected from Iran, 5 km west of Behshahr (36.692373 latitude, 53.475609 longitude, 8 m altitude). Subsequently seeds were maintained and increased at the Wheat Genetics Resource Center (WGRC) at Kansas State University, Manhattan, KS, United States. All the plants were grown in a 1:1 vermiculite:soil mixture in cone of 2 inch diameter. The seedlings at two leaf-stage were vernalized at 4°C for 6 weeks in growth chambers, after which they were grown in greenhouse at 20–25°C with a light period of 16 h. Since *Ae. tauschii* shatters at maturity, all the plants were individually enclosed in plastic covers before the onset of flowering to avoid loss of seeds.

To chemically mutagenize the seeds, Ethyl methanesulfonate (EMS) from Sigma Aldrich (Cat No. M0880-25G) was used as the mutagen. To determine the appropriate concentration of EMS, two rounds of tests were made. The target of first round was to optimize a dose of the EMS needed to achieve 40–60% survival among the M_1_ plants. Based on our prior experience with a diploid ‘A’ genome *Triticum monococcum* TILLING population (Rawat et al., 2012), first round of treatments included low concentrations of EMS solutions (0.15, 0.2, 0.24, and 0.27%). The protocol for mutagenesis was same as of Rawat et al. (2012). Briefly, 100 seeds of TA 2450 were soaked in 50 ml water in 250 ml glass flasks for 8 h of imbibition on a shaker at 100 rpm and then treated with five different doses (0, 0.15, 0.2, 0.24, and 0.27%) of EMS for 16 h on shaker at 75 rpm. The treated seeds were washed under running water for 8 h and then transplanted individually into cones. Observations were made 15 days after transplanting to estimate the survival frequency. However, the LD50 of these low treatments were found to be much lower than desirable. So, another round of dosage optimization was made with much higher concentrations this time (0.3, 0.4, 0.5, 0.6, and 0.7%) using the same method. Untreated controls were included in all the experiments to make valid comparisons of all the treatment dosages. EMS dose of 0.6% was found to provide 60% survival and was selected for treating a total of 5,300 TA-2450 seeds.

A total of 3,887 M_1_ plants were derived from the M_0_ EMS-treated seed and were allowed to self. Two thousand nine hundred and seventy M_1_ plants were fertile. A single M_2_ plant was grown from every M_1_ plant to prevent genetic redundancy. Tissue was collected, and the spikes cataloged at maturity for all fertile 2,656 M_2_ individuals of the TILLING population.

### Development of DNA Pools

Leaf tissue from all M_2_ individuals was collected at the four-leaf stage in 96-well blocks. The tissue was lyophilized and stored in -80°C until use. A set of 1,180 M_2_ plants was used for DNA extraction and subsequent characterization of the TILLING population. DNA was isolated using a Kingfisher Flex DNA extraction robot with Biosprint 96 plant DNA extraction kit (Qiagen, Valencia, CA, United States) according to the manufacturer’s instructions. DNA was quantified on a Nano-drop and normalized to 25 ng/μl in 96-well blocks. Subsequently, 4x pooling was done, combining four plates of DNA into one pool plate retaining the row and column identity of the samples. Two hundred microliters of normalized DNA from each pool member was combined. The mutants were cataloged, and their DNA was identified with a unique ID as Pool-Plate-Row-Column.

### TILLING for Genes of Interest

**FIGURE 2:**
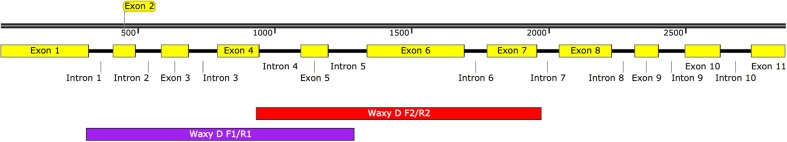
Position of primers used for TILLING *waxy* gene in the TILL-D resource.

*Waxy* gene, that encodes a granule-bound starch synthase protein required for the synthesis of amylose in starchy endosperms was used for characterizing mutation frequency of our TILLING population (Yamamori et al., 1994). Two primer pairs were used that covered Exons 2–5 and Exons 5–7. Figure 2 shows the position of primers used for the *waxy* gene. One more gene, *4-coumarate-CoA ligase 1* (*4CL1*), from lignin biosynthesis pathway, previously used in characterizing a diploid ‘A’ genome TILLING population (Rawat et al., 2012) was also used for the sake of making comparison with another diploid wheat species. List of primers used has been provided in Table 1.

**Table 1:** Primer sequences and product sizes of the primers used for TILLING.

Primer name	Gene	Sequence 5′–3′	Product size
Waxy_D_F1	*Waxy*	CCATGGCCGTAAGCTAGAC	978
Waxy_D_R1		CGCAAAATTGATATGCCTGTT	
Waxy_D_F2	*Waxy*	TGGGCCCTACGGTAAGATC	1039
Waxy_D_R2		GGGCTCGATGATGTACCAGG	
4CL1_CF	*4CL1*	AGAGTCCACCAAGAACACCATC	782
4CL1_CR		CTGGCTCTCAAGTCCTTCCTC	


### PCR, Cel-I Assays and Mutant Detection

The primers were used on pooled DNA for PCR amplification using Bioline MyTaq PCR kits (Bioline, Tauton, MA, United States) in 25 ul volume, using BioRad 100 thermocycler (BioRad, Hercules, CA, United States). All 1,180 pooled M_2_ individuals were screened for mutations in the waxy gene and 4CL1 gene. A touch down PCR profile (95°C–5 min, seven cycles of 95°C–1 min, 67–60°C-min with a decrease of 1°C per cycle, 72°C–2 min, followed by 30 cycles of 95°C–1 min, 60°C–1 min, 72°C–2 min, and a final extension of 72°C–7 min) was used. PCR products were subsequently denatured and slowly reannealed to form heteroduplexes between mismatched DNA (95°C–2 min, five cycles of 95°C–01 s, 95–85°C-1 min with a decrease of 2°C per cycle, and 60 cycles of 85–25°C–10 s. Cel-1 endonucelase was extracted from celery stalks following the protocol of Till et al. (2006) The homemade Cel-1 was tested for optimum activity with known mutants characterized previously. Two and a half μl of Cel-I was added to the heteroduplexed products and incubated at 45°C for 45 min. Reactions were stopped using 2.5 μl 0.5 M EDTA.

**FIGURE 3:**
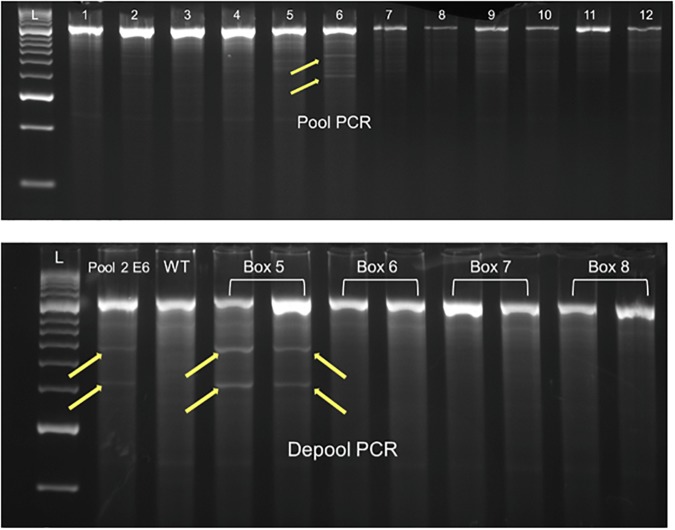
Mutant identification using Cel-1 assay and agarose-gel based platform.

The digested products were visualized on 2.5% agarose gels. Mutants could be identified as the products showing cleaved bands in addition to the full-length, uncleaved product (Figure 3). The total number of bases scanned was calculated by subtracting 20% of the product size, to take into account the primer base pairs and terminal regions that escape detection as has been done previously on various detection platforms (Slade et al., 2005; Dong et al., 2009; Rawat et al., 2012).

### Deconvolution and Sequencing of Mutants

Pools identified to carry mutation were deconvoluted using the same procedure as described above on the individual members of the pools. To identify the homo/heterozygosity of the mutant plants, PCR was performed with each constituent pool member in two copies, one with wild type *Ae. tauschii* DNA added, and another without it. After identifying the individual carrying the mutation, Sanger sequencing of the PCR product was done on an ABI3739xl (Applied Biosystems, Foster City, CA, United States) as per the manufacturer’s instructions. Provean scores were calculated for mis-sense mutations using PROVEAN protein webtool at http://provean.jcvi.org.

## Results

### Development of the TILL-D Population

**FIGURE 4:**
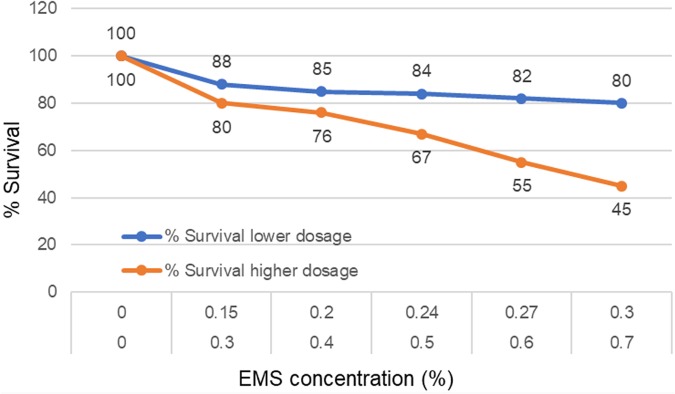
Dosage optimization with various EMS concentrations to find appropriate concentration providing optimum survival of mutagenized individuals.

Two rounds of dosage optimization experiments were conducted for developing the TILL-D population. The initial experiment was done with EMS concentrations of 0.15, 0.2, 0.24, 0.27, and 0.3%. However, the kill-rate, an indicator of the effectiveness of mutagenesis, was very low (Figure 4) with these concentrations. Next round of treatments included much higher concentrations (0.3, 0.4, 0.5, 0.6, and 0.7%) of EMS (Figure 4). Treatment with 0.6% EMS solution was found to provide an optimal kill-rate of 40% and was selected for the treatment of the entire batch of 5,300 seeds of *Ae. tauschii* accession TA2450. A total of 3,887 M_1_ plants were produced from the treatment. Out of these 2,970 plants set M_2_ seeds and were planted to produce the M_2_ population. However, 153 M_2_ seeds did not germinate, so leaf tissue could not be collected for these. One hundred and sixty-one M_2_ plants were sterile. Seeds could not be retrieved in M_3_ generation for both these types of mutants. The final size of the fertile TILL-D population that was cataloged at M_3_ generation was 2,656 individuals.

**FIGURE 5:**
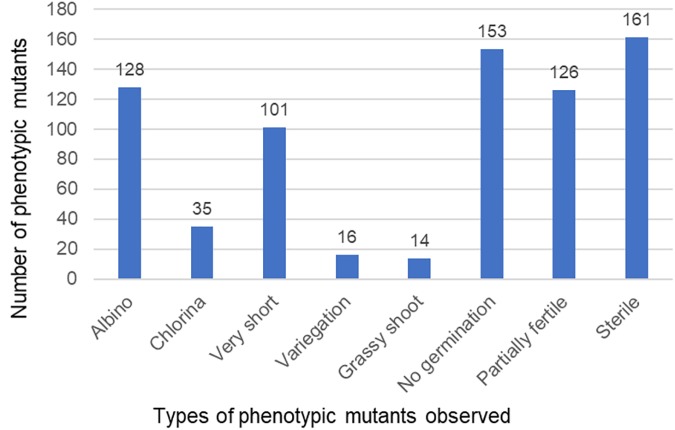
Number of phenotypic mutants observed in the TILLING population.

The M_2_ population showed phenotypic mutants such as albinos, chlorinas, very short, variegated, grass-like, and male sterile (Figure 1). Some M_2_ seeds did not germinate at all. Figure 5 shows the range of phenotypic mutants observed for the TILLING population. A total of 24.7% of the planted population showed phenotypic mutants.

### TILLING for Identifying Mutants

Since the goal of this work was to characterize the mutation frequency of the TILL-D resource, we selected the *waxy* gene that has been extensively used for characterizing various TILLING populations. Mutation frequencies for the *waxy* gene can be compared with known diploid, tetraploid, and hexaploid wheat TILLING populations (Slade et al., 2005; Dong et al., 2009; Rawat et al., 2012). Cel-1 based mutation detection for *waxy* gene was done with two primer pairs, waxy_D_F1+R1 and waxy_D_F2+R2, giving product sizes of 978 and 1039 bp, respectively. A total of seven mutants, four for waxy_D_F1+R1, and three for waxy_D_F2+R2 were identified after screening 230 and 245 kb, respectively. The mutation frequency of the TILL-D population using the *waxy* gene was found to be 1/70 kb. All the mutants identified for *waxy* were heterozygous.

TILLING was also performed on the gene *4CL1* using 4CL1_CF+CR primer pair, generating a product size of 782 bp. Two mutants were found after scanning a total of 185 kb, providing a mutation frequency of 1/92 kb for this gene. One of the *4CL1* mutants was homozygous, and the other had a heterozygous mutation.

The overall mutation frequency of the TILL-D population scanning a total of 661 kb using all the three primers was found to be 1/77 kb.

### Sequencing of the Mutants

All the mutants identified were G > A or C > T transitions, characteristic mutations produced by EMS treatments (Table 2). Out of the seven mutants identified for the *waxy* gene, three lied in the introns. Out of the remaining four, two were silent mutations that did not have any amino acid change, and the other two were mis-sense mutations that changed amino acid sequences. PROVEAN scores were calculated for the two mis-sense mutations and one was found to be deleterious (Table 2). The two mutations identified for the *4CL1* gene were both intronic.

**Table 2:** Details of the sequence variations found in the mutant individuals of the TILL-D population.

Primer set forward/reverse	Plant ID (Pool-Box-RowColumn)	Base pair change	Homo/heterozygous	Type of mutation	Amino acid change	Location of mutation	Provean score	Prediction
Waxy_D_F1+R1	2-8-E5	C > T	Hetero	Intronic	–	Intron 3	–	–
	2-5-E6	G > A	Hetero	Silent	Silent	Exon 4	–	–
	2-6-E2	C > T	Hetero	Intronic	–	Intron 3	–	–
	3-9-B5	C > T	Hetero	Silent	Silent	Exon 4	–	–
Waxy_D_F2+R2	3-9-H4	C > T	Hetero	Intronic	–	Intron 6	–	–
	2-7-D2	G > A	Hetero	Mis-sense	G348D	Exon 6	-6.098	Deleterious
	2-7-G9	C > T	Hetero	Mis-sense	A376T	Exon 7	-1.230	Neutral
4CL1_CR+CF	3-10-E10	C > T	Homo	Intronic	–	Inton 1	–	–
	1-4-F12	A > G	Hetero	Intronic	–	Intron 2	–	–


## Discussion

*Aegilops tauschii*, the diploid D genome donor of hexaploid wheat, has been extensively used as a rich source of useful genes of biotic and abiotic stress tolerance, quality improvement and yield parameters for wheat improvement (Gill et al., 1986b; Dhaliwal et al., 1991; Rasheed et al., 2018). With the genome sequence availability of *Ae. tauschii* (Luo et al., 2017), it will be comparatively easier now to map the genes of interest derived from it. The TILL-D population provides an array of allelic variants that will be useful for both forward and reverse genetics of desirable traits. Nevertheless, developing mutagenized populations in diploid plants is a delicate exercise, as too low concentration of the mutagen is not very effective in creating sufficient mutations in the genome, and a high concentration treatment with the mutagen is detrimental to the survival of the plants. Polyploids such as durum and bread wheat, however, can tolerate higher doses of mutations because of genome buffering (Comai, 2005; Feldman and Levy, 2012). EMS concentrations ranging from 0.6 to 1% have been used in developing polyploid wheat TILLING populations (Sestili et al., 2009; Uauy et al., 2009; Chen et al., 2012; Rawat et al., 2016). On the other hand, Rawat et al. (2012) developed a diploid ‘A’ genome wheat *Triticum monococcum* TILLING population using a concentration of 0.25% EMS, achieving the mutation rate of 1/92 kb. In this work, however, *Ae. tauschii* apparently tolerated a much higher concentration of mutagen (0.6% EMS) as compared with *T. monococcum.* The tolerance of *Ae. tauschii* to higher mutagen concentration than *T. monococcum* is interesting and the exact reason for this observation is not known, as no prior documented report on mutagenesis of wild relatives of crop plants is available. *Ae. tauschii*, being a wild relative may be hardier than cultivated einkorn wheat to tolerate mutations, that is also supported by the high mutation frequency of the TILL-D population.

Mutation frequency of the TILL-D population was found to be quite high (1/77 kb) for a diploid plant. Diploid TILLING populations of Arabidopsis (1/300 kb, Greene et al., 2003; 1/170 kb Till et al., 2003), sorghum (1/526 kb, Xin et al., 2008), rice (1/294 kb, Till et al., 2007; 1/135 kb, Suzuki et al., 2008), barley (1/1000 kb, Caldwell et al., 2004; 1/374 kb, Talame et al., 2008), and einkorn wheat (1/92 kb Rawat et al., 2012) have been reported to have much lower mutation frequencies. Massa et al. (2011) studied the gene space dynamics during the evolution of diploid *Ae. tauschii, Brachypodium distachyon*, rice, and sorghum, and found that due to widespread gene duplication and very low gene deletion events in *Ae. tauschii*, the overall gene number increased by 7,813 genes from the common ancestor. The rate of gene duplications and insertions over the past 45–60 million years was the highest and rate of gene deletions was the lowest in *Ae. tauschii* among the four diploid genomes relative to the common ancestor. This gene redundancy and higher gene content may be involved in making *Ae. tauschii* more tolerant of mutagenesis events in its genome. However, an exact explanation of this observation should be investigated further.

Different mutation frequencies are reported for different genes in wheat (Slade et al., 2005; Uauy et al., 2009). Therefore, we selected *waxy* gene for calculating the mutation frequency of *Ae. tauschii* TILLING population, to make valid comparisons with other diploid, tetraploid, and hexaploid wheat TILLING populations. A mutation frequency of 1/70 kb was observed for *waxy* gene in our TILL-D population, whereas tetraploid and hexaploid wheat TILLING populations have been reported to have much higher mutation frequencies for the same gene. Slade et al. (2005) reported mutation frequencies of 1/24 and 1/40 kb for *waxy* gene in TILLING populations of hexaploid wheat variety ‘Express’ and tetraploid wheat variety ‘Kronos,’ respectively. TILLING populations of hexaploid wheat varieties QAL2000 and Ventura were found to have mutation frequencies of 1/23 and 1/36 kb, respectively (Dong et al., 2009). The order of mutation frequencies of diploid < tetraploid < hexaploid with the same gene is as per expectations because of genome buffering that allows polyploids to tolerate higher number of variations per genome. It is an evolutionary advantage for the plants, but at the same time, makes it difficult to relate functions with genes (Comai, 2005; Dubcovsky and Dvorak, 2007; Rawat et al., 2012). Therefore, diploid genotypes provide resources for straightforward gene validation studies, allele mining, and quick gene discoveries.

## Conclusion

The *Ae. tauschii* TILLING population developed will be a useful genetic resource for wheat improvement. Coupled with the *Ae. tauschii* genome sequence, it will provide a platform for allele mining and gene discovery in wheat. For gene cloning experiments of ‘D’ genome mapped genes it provides a permanent reverse genetic resource for gene function validation. With a high mutation frequency of 1 mutation every 77 kb, it is a rich permanent collection of variant alleles that can be exploited for either reverse genetics strategies or forward genetic screens to sift useful traits. Hexaploid TILLING populations in wheat have been found to have much higher mutation frequencies, but to see a phenotype due to a variant allele it is important to create mutation on all functional homoeologous (A, B, and D genome) copies of the gene. Having a mutant allele in a diploid will express the phenotype readily, leading to quick gene discovery. Such information can be used to generate mutants in bread wheat using hexaploid TILLING populations or gene editing approaches. Seed of the TILL-D population are being increased and will be made available after the M_4_ generation to users upon request.

## Author Contributions

NR and VT conceived the study, planned the experiments, and were primarily responsible for drafting and revising the manuscript with contributions from co-authors. AS, LS, and AM performed DNA extraction, pooling, PCRs, and *Cel*-I extraction and sequence analysis. NR, GL, DW, BG, and SL developed the mutant population. DW phenotyped the population, collected and archived the tissue and seeds stocks. All authors read and approved the final manuscript.

## Conflict of Interest Statement

The authors declare that the research was conducted in the absence of any commercial or financial relationships that could be construed as a potential conflict of interest.
